# Multiple abscesses in the lower extremities caused by Trichophyton rubrum

**DOI:** 10.1186/s12879-019-3897-3

**Published:** 2019-03-20

**Authors:** Yeqin Dai, Xiujiao Xia, Hong Shen

**Affiliations:** Department of Dermatology, Hangzhou Third Hospital, Zhejiang University of Traditional Chinese Medicine, Hangzhou, 310009 People’s Republic of China

**Keywords:** Dermatophyte abscess, Immunocompromised, Trichophyton rubrum

## Abstract

**Background:**

Dermatophytes are keratinophilic fungi, that usually infect the hair, stratum corneum, and nails. However, dermatophytes occasionally invade the dermis, subcutaneous tissues, and internal organs, resulting in a condition called deep dermatophytosis. We report a case of an unusual presentation of *Trichophyton rubrum* infection causing multiple fungal abscesses in the lower extremities of an immunocompromised patient.

**Case presentation:**

A 66-year-old male who had been receiving immunosuppressive drugs for 7 years developed numerous subcutaneous nodules in the lower extremities. The yellow purulent fluid obtained from the cyst was positive for *T. rubrum*. Topical bifonazole cream was effective for tinea pedis, but oral Sporanox 400 mg/day was discontinued after 2 months because the patient died from pneumonia after hospitalization for a lumbar fracture.

**Conclusions:**

Although deep dermatophytosis is very rare, dermatomycosis should be considered in any examination of patients who are receiving immunosuppressive drugs. Fungi can enter the bloodstream and disseminate to distant major organs, including the lymph nodes, liver, brain, and bone, which often causes systemic infections that can be fatal.

## Background

Dermatophytes are keratinophilic fungi that usually infect the hair, stratum corneum, and nails. However, dermatophytes occasionally invade the dermis, subcutaneous tissues, and internal organs, resulting in a condition called deep dermatophytosis. We report a case of an unusual presentation of *Trichophyton rubrum* infection causing multiple fungal abscesses in the lower extremities of an immunocompromised patient.

## Case presentation

**Fig. 1:**
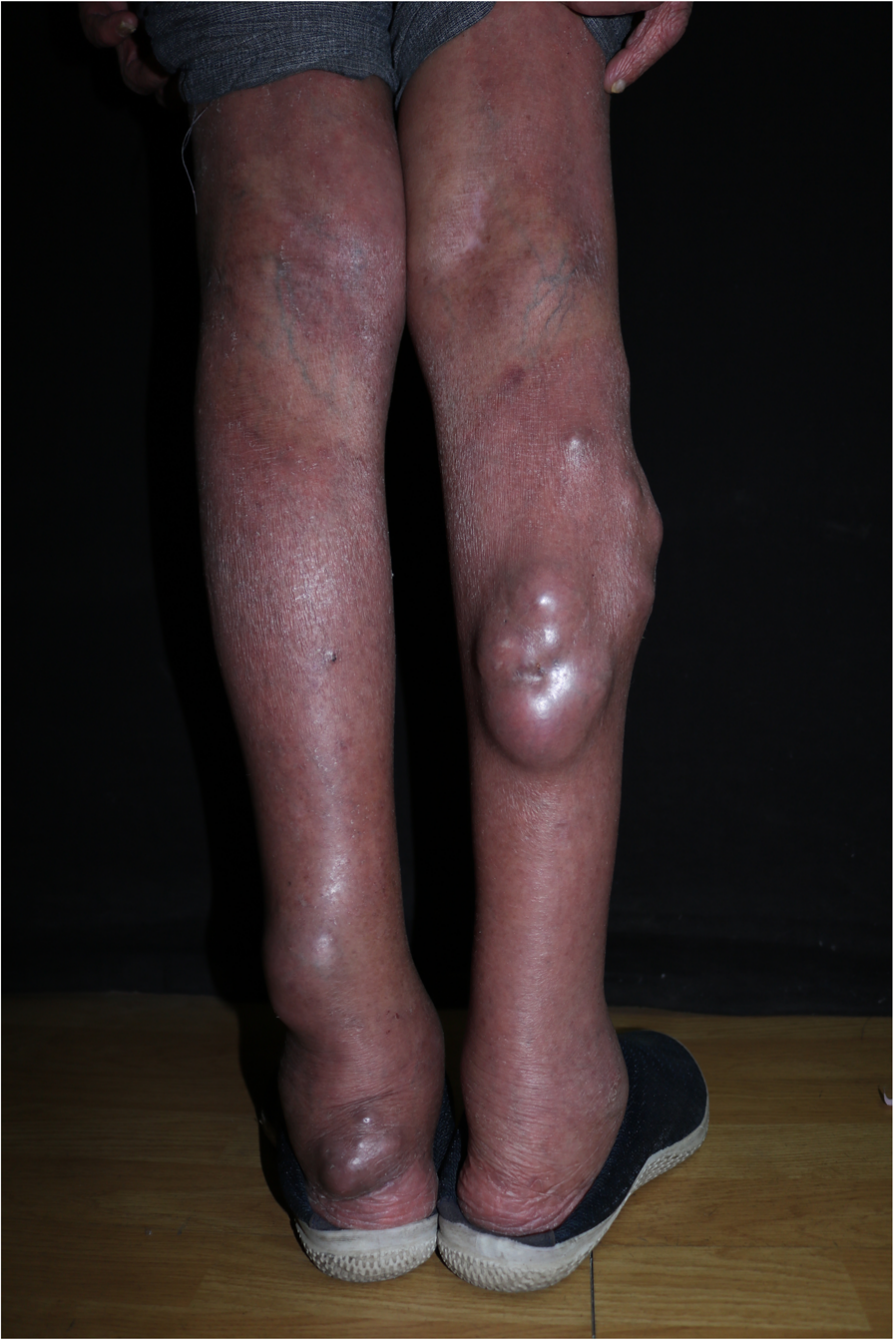
Numerous well-demarcated, elastic, soft, partially fluctuant, multilocular subcutaneous cysts measuring 2–10 cm in diameter

A 66-year-old peasant from Anhui Province developed numerous subcutaneous nodules in the lower extremities without any trauma, and the nodules gradually grew over 2 years. Bullous pemphigoid was diagnosed at the age of 59 years while he was in the care of the Department of Dermatology, Shanghai Huashan Hospital (2010), and was treated with steroid therapy. A physical examination revealed erythema, blisters and itching all over the body. The doctor prescribed oral methylprednisolone 24 mg/d. Two years later, when the symptoms had subsided, he remained on methylprednisolone 12 mg/d and often suffered from severe back and thoracic pain. One year ago, his dose decreased to methylprednisolone 6 mg/d. In July 2017, he came to our department because of numerous subcutaneous nodules in the lower extremities. A physical examination revealed a blood pressure of 127/77 mmHg, respiratory rate of 18 breaths per minute, pulse rate of 75 beats per minute, temperature of 37 degrees Celsius, and oxygen saturation of 100% on room air. Moreover, the examination revealed numerous well-demarcated, elastic, soft, partially fluctuant, multilocular subcutaneous cysts measuring 2–10 cm in diameter (Fig. 1). There were some red spots and scales on the lower extremities. The rest of the physical examination was unremarkable.

There was no redness, tenderness, fistula formation, or purulent discharge. Clinical manifestations were not apparent on the surface skin of the cyst, and a small specimen of the scales was obtained by scraping the skin with a scalpel. A KOH preparation test of the scales revealed septated hyphae.

**Fig. 2:**
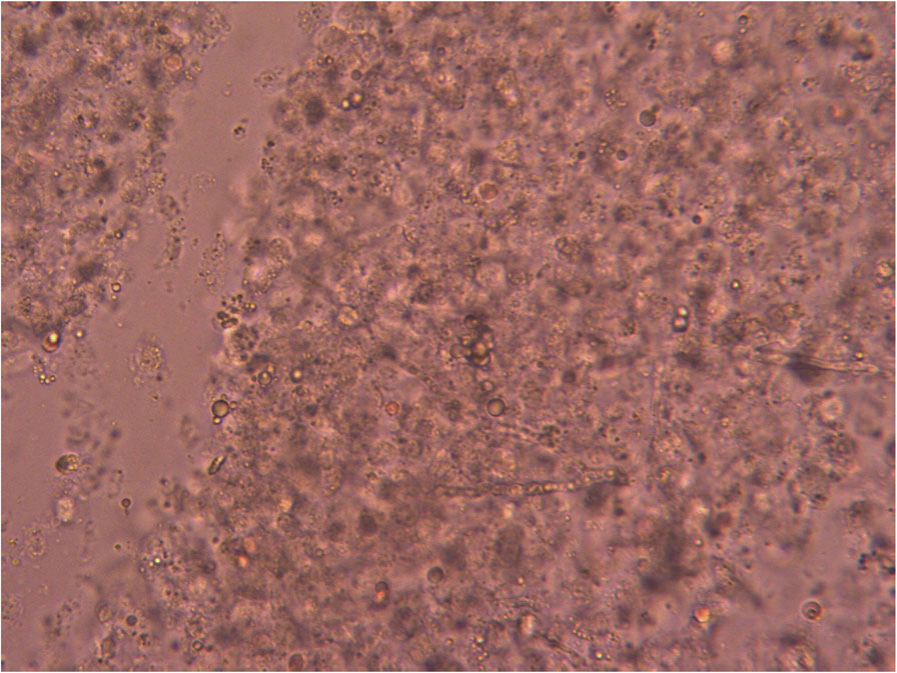
A KOH preparation test of the purulent fluid revealed a large number of septated hyphae

A large amount of yellow purulent fluid was obtained from the cyst by needle aspiration, and a KOH preparation test of this fluid revealed a large number of septated hyphae (Fig. 2). Bacterial and fungal cultures of the pus were performed to discriminate true fungal infection from contamination. The bacterial culture of the pus was negative, and the fungal culture yielded *T. rubrum*. Laboratory tests revealed a white blood cell count of 8300/μl (normal range, 3900-9700/μl), 20.0 mg/dl blood urea nitrogen (9–21 mg/dl), 0.95 mg/dl creatine (0.6–1.2 mg/dl), and 1.7 mg/dl C-reactive protein (< 0.2 mg/dl). Abdominal ultrasonography revealed a small cyst in the liver. The rest of the ultrasonography examination was unremarkable.

The ultrasound examination revealed several cystic nodules of different sizes in the subcutaneous soft tissue, some of which were fused with each other, with dotted echoes floating inside.

**Fig. 3:**
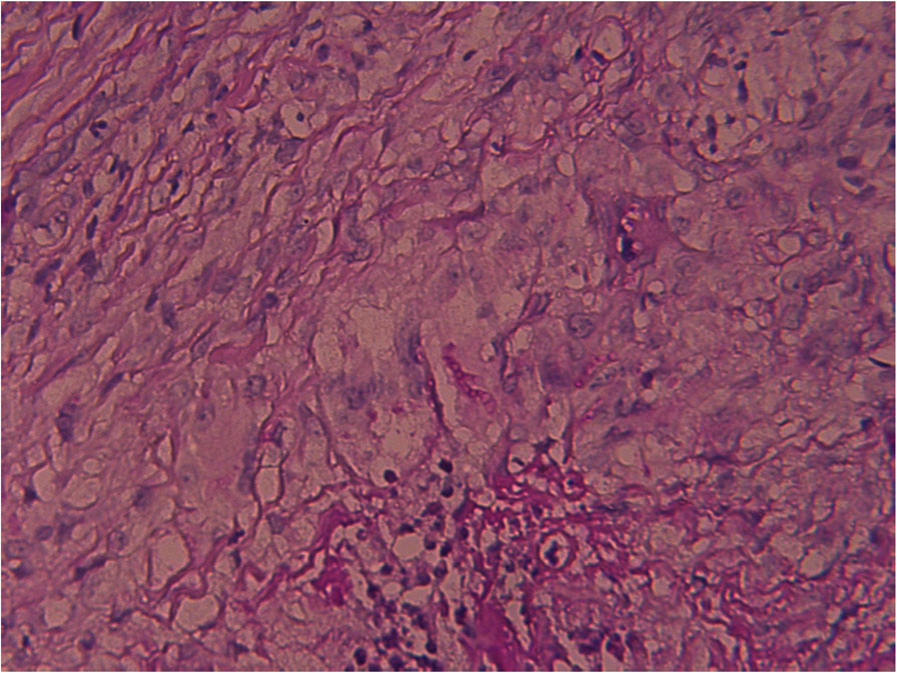
Pathological examination showed a multilocular cyst, in which the cyst wall formed granulomas composed of giant cells, histiocytes, and lymphocytes and the interior of the cyst formed abscesses composed of neutrophils

**Fig. 4:**
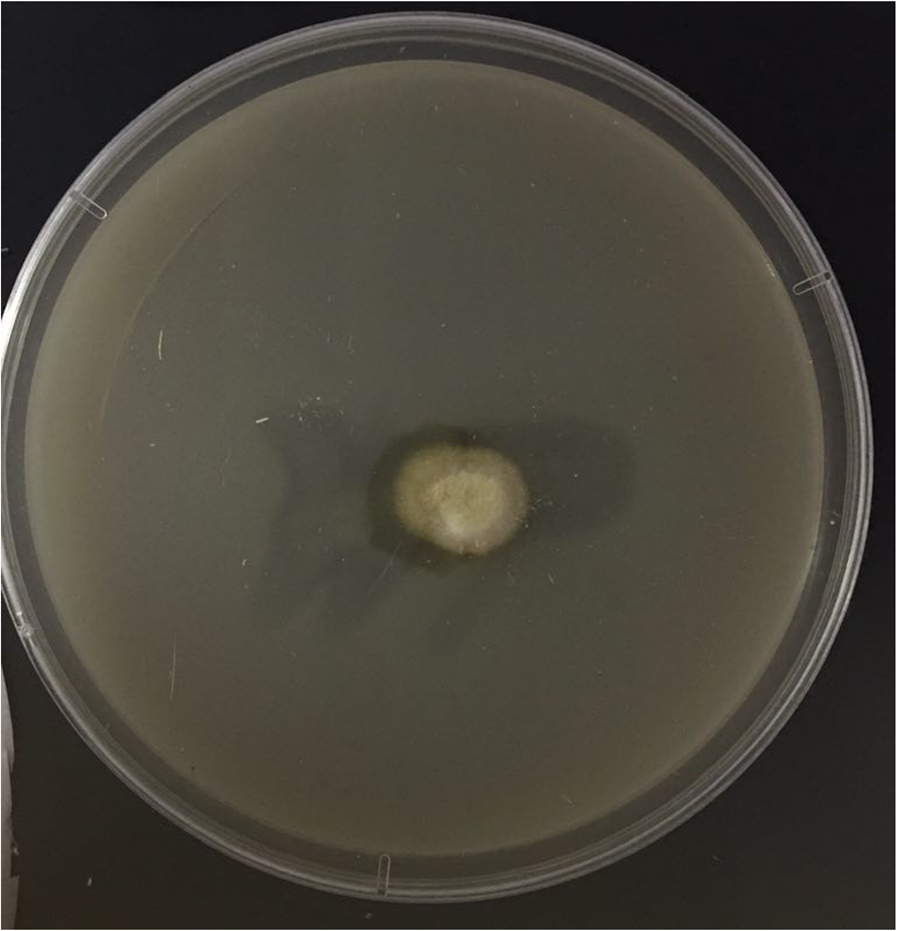
Fungal cultures of the scales and abscesses yielded slightly raised, yellowish chorionic colonies

The pathological examination revealed a multilocular cyst; the cyst wall formed granulomas composed of giant cells, histiocytes, and lymphocytes, and the interior of the cyst formed an abscess composed of neutrophils (Fig. 3). Fungal cultures of the scales and abscesses yielded slightly raised, yellowish chorionic colonies on Sabouraud dextrose agar (Fig. 4). A subculture of the isolate was submitted to Shanghai Sangong Biotech Co. Ltd., for sequencing. A BLAST search of the GenBank database showed that the internal transcribed spacer region of nuclear ribosomal DNA from the isolated pathogen had 99% homology with *T. rubrum* (GenBank accession no. KP326579.1). Finally, the isolate was identified as *T. rubrum* based on the fungus morphology and DNA sequencing results.

The patient was diagnosed with recurrent dermatophyte abscesses caused by *T. rubrum*, and oral itraconazole 200 mg once daily was started, along with bifonazole cream. Two months later, on 18 September 2017, he was hospitalized in the Department of Orthopedics, HangZhou Third Hospital, for a lumbar fracture. Two days later, he developed a fever of 39 °C and was diagnosed with pneumonia by computed tomography imaging of the lungs. The patient’s kin made a decision to allow the patient to forgo treatment and die at home.

## Discussion and conclusions

Dermatophytes are common fungal pathogens that mainly cause superficial infections of the skin, nails, and hair. However, deeper dermis and subcutaneous dermatophyte infections can occur in patients with compromised immune systems due to solid organ transplantation, hematological malignancy, immunosuppressive therapy, or congenital immune deficiency. In these cases, fungi can enter the bloodstream and disseminate to distant major organs, including the lymph nodes, liver, brain, and bone, often causing systemic infections that can be fatal.

The lesions most frequently affect the lower extremities, including the buttocks and groin, although they can occur anywhere on the body. Deep dermal dermatophytosis tends to present as multiple rather than solitary nodules, and the patients frequently have superficial dermatophytosis as well. The most common pathogen for deep dermal dermatophytosis is *T. rubrum*, but other causative pathogens include *T. violaceum*, *T. mentagrophytes*, *Microsporum canis*, *M. ferrugineum*, and *T. verrucosum*. An elevation of beta-D glucan was noted, which may suggest an invasive fungal infection.

Deep dermatophytosis is very rare, with only 100 cases reported in the literature [1]. The classification of deep dermatophytosis has not been established due to the diversity of its clinical manifestations [2]. In deep dermatophytosis, deep lesions are usually accompanied by superficial dermatophytosis. Fukushiro [3] categorized deep dermatophytosis into the following four clinical entities: (1) dermatophyte granuloma, subcategorized as (1a) localized disease, in which subcutaneous nodule formation is accompanied by preexisting superficial dermatophytosis at selected skin sites, or (1b) systemic disease, in which subcutaneous nodule formation is accompanied by diffuse superficial dermatophytosis that may disseminate to internal organs and cause death (granuloma formation, not abscesses, is seen in the nodules in both subgroups); (2) nodular granulomatous perifolliculitis of the legs, in which multiple chronic nodules surrounding hair follicles are observed unilaterally in the lower leg, representing a variant form of the clinical entity indicated in 1a; (3) dermatophyte abscess, consisting mainly of abscesses containing dermatophytes in the dermis and/or subcutis; and (4) dermatophytic mycetoma, for which the clinical manifestations include the formation of a sinus tract that discharges exudate containing dermatophytic granules.

Si-Hyun Kim et al. [4] reported a rare case of dermatophyte abscesses caused by *T. rubrum* in an immunocompromised patient without pre-existing superficial dermatophytosis. A lack of experience with dermatophytosis could make clinicians underestimate the significance of positive dermatophyte fungal cultures obtained from deep soft tissue. Even without any superficial dermatophytosis lesions, fungi should be considered as a possible cause of deep soft tissue abscesses in immunocompromised patients, and fungal and bacterial cultures should be performed for these patients.

Utako Okata-Karigane et al. [5] first reported a deep dermal dermatophytosis that mimicked lymphadenitis. The standard treatment for deep dermal dermatophytosis has not been established, but systemic antifungal therapy is generally selected, and oral terbinafine or itraconazole are effective. When the lesion forms a well-demarcated, subcutaneous nodule, the combination of resection and systemic antifungal therapy might be useful.

Badali H [6] and Rezaei-Matehkolaei A et al. [7] confirmed that terbinafine is an excellent agent for the treatment of dermatophytosis due to *T. rubrum*. Claire Rouzaud et al. [8] clarified that the diagnosis of severe invasive or extensive dermatophytosis without clearly identifiable risk factors should prompt screening for inherited immunodeficiencies, such as CARD9 deficiency. Afsane Vaezi et al. [9] evaluated the frequency, geographic distribution and nature of mutations in patients with CARD9 deficiency. They identified 60 patients with 24 mutations and different fungal infections.The presence of the homozygous (HMZ) p.Q295X (c.883C > T) and HMZ p.Q289X (c.865C > T) mutations were associated with an elevated risk of candidiasis (OR: 1.6; 95% CI: 1.18–2.15; p = 0.004) and dermatophytosis (OR: 1.85; 95% CI: 1.47–2.37; p < 0.001), respectively. The geographical distribution differed, showing that the main mutations in African patients were different Asian patients; HMZ p.Q289X (c.865C > T) and HMZ p.Q295X (c.865C > T) accounted for 75% and 37.9% of the African and Asian cases, respectively. The spectrum of CARD9 mutations in Asian patients was higher than in African. Asia is the most populous continent in the world and may have a greater genetic burden resulting in more patients with severe fungal infections. The presence of a high diversity of mutations revealing 24 distinct variations among 60 patients emphasize that the unique genetic alteration in CARD9 gene may be associated with certain geographical areas.The prevalence of these severe forms may be underestimated. Prompt and adequate treatment of superficial dermatophytosis when starting immunosuppressive treatment is important for preventing the development of more severe forms of the disease.

Our patient presented with symptoms similar to those of dermatophyte abscesses, showing mainly abscesses containing dermatophytes in the dermis and/or subcutis. There were numerous subcutaneous nodules containing purulent fluid in the lower extremities. There was no redness, tenderness, fistula formation, or purulent discharge. He had no fever and no relevant family history. Abdominal ultrasonography revealed a small cyst in the liver, but the rest of the ultrasonography examination was unremarkable. However, he often suffered from severe back and thoracic pain, which may be side effects of the immunosuppressive drugs. However, when he was hospitalized for a lumbar fracture, he developed a fever of 39 °C and was diagnosed with pneumonia upon computed tomography imaging of the lungs. It is a pity that the patient’s kin made the decision to allow him to forgo treatment and die at home. Thus, dermatomycosis should be considered in any examination of patients who are receiving immunosuppressive drugs. Fungi can enter the bloodstream and disseminate to distant major organs, including the lymph nodes, liver, brain, and bone, often causing systemic infections that can be fatal.

## Abbreviations

BLAST: Baseline artifact suppression technique
DNA: Deoxyribonucleic acid
KOH: Potassium hydroxide
